# Deep learning solutions for smart city challenges in urban development

**DOI:** 10.1038/s41598-024-55928-3

**Published:** 2024-03-02

**Authors:** Pengjun Wu, Zhanzhi Zhang, Xueyi Peng, Ran Wang

**Affiliations:** 1 School of Plastic Arts, Daegu University, Gyeongsan, Gyeongsangbukdo 38453 South Korea; 2 College of Art and Design, Southwest Forestry University, Kunming, 650224 Yunnan China; 3 Sichuan Energy Construction Group Design and Research Institute, Chengdu, 610011 Sichuan China; 4 China Construction Eighth Engineering Division Corp, LTD, Wuhan, 430000 Hubei China

**Keywords:** Smart cities, Deep learning, Bayesian regularization, Neural network, Planning, Urban infrastructure, Transportation management, Sustainability, Computational science, Computer science

## Abstract

In the realm of urban planning, the integration of deep learning technologies has emerged as a transformative force, promising to revolutionize the way cities are designed, managed, and optimized. This research embarks on a multifaceted exploration that combines the power of deep learning with Bayesian regularization techniques to enhance the performance and reliability of neural networks tailored for urban planning applications. Deep learning, characterized by its ability to extract complex patterns from vast urban datasets, has the potential to offer unprecedented insights into urban dynamics, transportation networks, and environmental sustainability. However, the complexity of these models often leads to challenges such as overfitting and limited interpretability. To address these issues, Bayesian regularization methods are employed to imbue neural networks with a principled framework that enhances generalization while quantifying predictive uncertainty. This research unfolds with the practical implementation of Bayesian regularization within neural networks, focusing on applications ranging from traffic prediction, urban infrastructure, data privacy, safety and security. By integrating Bayesian regularization, the aim is to, not only improve model performance in terms of accuracy and reliability but also to provide planners and decision-makers with probabilistic insights into the outcomes of various urban interventions. In tandem with quantitative assessments, graphical analysis is wielded as a crucial tool to visualize the inner workings of deep learning models in the context of urban planning. Through graphical representations, network visualizations, and decision boundary analysis, we uncover how Bayesian regularization influences neural network architecture and enhances interpretability.

## Introduction

In the wake of unprecedented global urbanization, the concept of the smart city has emerged as a transformative force reshaping the urban landscape. Urban areas are no longer mere conglomerations of buildings and infrastructure; they have evolved into complex ecosystems where data and technology converge to create more efficient, sustainable, and livable environments. The integration of digital innovation into urban planning and management is at the core of this transformation, and among the most prominent technologies driving this evolution are deep learning and neural networks. As we peer into the future, the need for innovative urban solutions becomes increasingly evident. By 2050, 68% of the world's population will reside in cities, according to the United Nations^1^. Numerous issues, including transportation congestion, energy consumption, public safety, and environmental sustainability, are brought on by this extraordinary urban expansion. The pace and scope of urbanization provide challenges for conventional urban planning paradigms^2^.

Technology has become a crucial driver for advancement in this continuously changing urban environment. In this urban transformation, deep learning, a branch of artificial intelligence (AI), has become a key technology. Deep learning algorithms, which are modeled after the neural networks in the human brain, have shown to be very adept at digesting large information, spotting minute patterns, and generating predictions that were previously unthinkable. Its inclusion in urban development and planning has sparked ground-breaking inventions and noticeable advancements in city living. Deep learning's importance in the creation of smart cities must be understood in light of the abundance of research and application that has paved the way. Previous research has provided priceless insights and shown the possibility of paradigm-shifting transformation. For instance, Anguita et al.^3^ demonstrated the effectiveness of neural networks in traffic prediction, offering the promise of smoother, more efficient urban mobility.

Furthermore, Nabavi et al.^4^ explored the optimization of energy consumption patterns in smart grids through deep learning, contributing to more sustainable urban energy practices. Building on the foundations established by these and other pioneering works, this article embarks on an extensive exploration of deep learning’s pivotal role in urban planning and smart city development. We venture into the multifaceted realms of urban life and infrastructure, where deep learning’s prowess is being harnessed to address the most pressing challenges of our time. The subsequent sections of this article will delve into the specifics of this transformative journey. We will elucidate how deep learning operates as a catalytic force in revolutionizing urban planning. We will dig into the complex network of approaches and data sources that drive this technological revolution, emphasizing how data, algorithms, and results interact in complex ways. We are going to set out on a thorough tour across several areas, revealing the range of applications that range from waste management and energy efficiency to traffic control and public safety. While addressing the difficulties brought on by this digital transformation, we will also explore the possibilities it offers for creating cities that are more fair, sustainable, and resilient. The awareness that deep learning is not just a technological advancement but also a crucial catalyst for fostering a smarter, more connected, and inclusive urban future is the common theme running through this story. This essay seeks to provide readers a sophisticated knowledge of how deep learning and neural networks are influencing the cities we live in and the cities we desire to develop, serving as a full roadmap for navigating this revolutionary journey.

## Related works

Deep learning will play a crucial part in the development of smart cities and urban planning, according to a plethora of studies. Anguita et al.'s^3^ demonstration of the use of neural networks for traffic prediction was a significant development that revealed possible remedies for reducing traffic congestion and improving urban mobility. Taking this as a foundation, Labiadh et al.^5^ carried out a thorough analysis of deep learning methods aimed at reducing energy consumption in buildings, offering significant insights into the sustainable energy practices needed in urban settings. Deep learning has made ground-breaking advances in the fields of surveillance and public safety. Convolutional neural networks (CNNs) have made substantial advancements in real-time threat identification capabilities, according to Sandler et al.^6^, who investigated their effectiveness for object detection in surveillance settings. Chandan et al.^7^ made noteworthy contributions to the intersection of deep learning and video analytics, expanding the discourse on advanced surveillance systems and their impact on public safety in metropolitan areas. By examining the combination of these technologies, the authors likely provided insights into how deep learning enhances the capabilities of video analytics, resulting in more sophisticated and effective surveillance mechanisms. The research may have delved into the specific applications of deep learning in surveillance, showcasing how the technology enables the identification, tracking, and analysis of objects or activities in video footage. These advancements contribute to the development of intelligent surveillance systems that can enhance situational awareness, aid in crime prevention, and improve emergency response in urban settings.

Fuentes et al.^8^ delved into the potential of deep learning algorithms in environmental monitoring, offering innovative solutions for tackling urban sustainability challenges. In the domain of urban mobility, Lv et al.^9^ explored the use of recurrent neural networks (RNNs) for short-term traffic flow prediction, shedding light on strategies for more efficient transportation management. Y. Li et al. introduce a Federated Deep Intrusion Anomaly (FDIA) detection method that draws inspiration from federated learning and incorporates secure federated deep learning. The proposed approach combines the capabilities of Transformer, federated learning, and the Paillier cryptosystem. In this method, the Transformer functions as a detector deployed at edge nodes, exploring the relationships between individual electrical quantities through its multi-head self-attention mechanism. Through a federated learning framework, the approach collaboratively trains a detection model using data from all nodes while maintaining data privacy by keeping the data local during training. To enhance the security of federated learning, the authors design a secure federated learning scheme by integrating the Paillier cryptosystem with federated learning^10^. Li introduces a forecasting scheme, termed Federated Deep Reinforcement Learning (FedDRL), for ultra-short-term wind power forecasting, combining federated learning and deep reinforcement learning (DRL). Initially, the paper employs the deep deterministic policy gradient (DDPG) algorithm as the foundational forecasting model to enhance prediction accuracy. Subsequently, the DDPG forecasting model is integrated into the federated learning framework. This design of FedDRL enables the acquisition of a precise prediction model in a decentralized manner by sharing model parameters instead of divulging private data, thereby mitigating concerns related to sensitive privacy issues^11^. Meanwhile, Shabestary et al.^12^ focused on the integration of deep learning with traffic signal control, advancing the optimization of urban mobility and traffic flow. Waste management, an ever-pressing urban challenge, saw promising developments with the work of Adedeji et al.^13^. They explored the potential of deep learning for waste classification, a crucial aspect of efficient waste management in urban areas. Moreover, the role of urban green spaces and their significance in urban planning was elucidated by Rojas-Rueda et al.^14^, who applied neural networks to analyze and prioritize green infrastructure within cities. Deep learning’s influence extended to urban health and well-being as well. Yuan Li presents a Power Demand–Supply Cooperative Responding (PDSCR) strategy that operates on both day-ahead and intraday time scales. The day-ahead PDSCR establishes a long-term strategy to address anticipated trends in Renewable Energy (RE) supply. However, recognizing that this long-term scheme may not be optimal when facing unpredictable RE fluctuations on an intraday basis, an intraday PDSCR is introduced. The intraday approach formulates a profit-driven cooperation strategy to effectively tackle the challenges posed by intraday RE fluctuations^15^. Islam et al.^16^ delved into its applications within the healthcare sector in smart cities, focusing on patient monitoring and health analytics. Infrastructure maintenance, a critical component of urban resilience, was examined by Best et al.^17^, who explored predictive maintenance of essential urban infrastructure using deep learning techniques. Nijkamp et al. delved into the theme of governance in the age of deep learning and smart cities, as explored in their work^18^. Their research underscores the critical significance of ethical considerations and policy frameworks in shaping the trajectory of smart cities. In the context of deep learning technologies, which play a pivotal role in the intelligence and automation of smart cities, ethical considerations become paramount. The authors likely discussed the potential impact of advanced technologies on urban governance, emphasizing the need for responsible practices to ensure that technological advancements align with societal values and norms.

Collectively, these studies provide a rich tapestry of insights, methodologies, and applications that underscore the transformative potential of deep learning and neural networks in urban planning and smart city development. They serve as a vital foundation for the exploration and discussion of these transformative technologies in our research. In the realm of smart cities, research often overlooks interdisciplinary approaches that could harmonize various urban domains, from traffic management to energy efficiency and public safety. Bridging these domains through interdisciplinary research could lead to more comprehensive and effective smart city solutions. Data privacy and security in smart cities represent a critical research gap. While data collection and analysis are emphasized, there’s a need for deeper exploration of robust data encryption, secure data sharing, and ethical considerations to protect citizens’ privacy in connected urban environments. Real-time decision-making systems within smart cities require further investigation. While AI excels in prediction, the translation of AI-driven insights into immediate actions for urban governance deserves attention for creating responsive urban environments. Sustainability metrics beyond energy efficiency need development. Establishing comprehensive measures for social, economic, and environmental sustainability is crucial for guiding sustainable urban development. Understanding the scalability and generalizability of smart city solutions across diverse regions and contexts is a research gap essential for creating adaptable technologies. Effective community engagement in shaping smart cities, facilitated by technology and AI, remains underexplored, emphasizing the importance of citizen-centric initiatives. There is a crucial need for research focused on bolstering urban resilience and disaster readiness through the application of AI and deep learning, especially given the escalating frequency of natural disasters. Bridging the digital divide to ensure affordability and inclusivity in smart city technologies is an urgent gap that requires attention, aiming to provide equitable access to technological advancements. It is imperative to explore the long-term effects, both positive and negative, of smart city technologies to guide sustainable urban development effectively. The development of extensive policy frameworks is vital for the responsible adoption and regulation of AI in smart cities, addressing legal and ethical intricacies while simultaneously fostering innovation.

To address interdisciplinary challenges, Artificial neural network (ANNs) can be employed to develop integrated models that span multiple urban domains, promoting synergy between urban planning and technology. In terms of data privacy and security, ANNs can play a crucial role by aiding the development of encryption methods and real-time anomaly detection systems, enhancing overall data protection in smart cities. For real-time decision-making, ANNs can swiftly analyze streaming data and offer immediate insights, enabling proactive responses to dynamic urban conditions. In creating comprehensive sustainability metrics, ANNs can assess a city’s performance across economic, environmental, and social dimensions, aiding policymakers in sustainable urban development. To enhance scalability and generalization, ANNs can adapt to different urban contexts and datasets, promoting the applicability of smart city solutions across diverse regions. Community engagement can be facilitated through neural network-driven platforms that gather citizen feedback and promote inclusivity in urban planning. Regarding disaster preparedness, ANNs can create predictive models that utilize historical and real-time data for early warnings and informed emergency responses. In ensuring affordability and inclusivity, ANNs can help optimize resource allocation, bridging the digital divide and making smart city technologies accessible to all. For long-term impact assessment, ANNs can analyze historical data and model future scenarios, aiding policymakers in making informed choices. Lastly, ANNs can assess policy effectiveness, guiding the development of comprehensive policy frameworks for responsible AI adoption in smart cities.

## Deep learning applications in smart cities

Deep learning techniques have become pivotal tools in tackling the complex challenges associated with smart city development. In the specific domain of traffic management and optimization, Duan et al.^9^ have showcased the effectiveness of recurrent neural networks (RNNs) in addressing short-term traffic flow prediction. This research likely involves the utilization of RNNs, a type of neural network designed to handle sequential data, for accurately forecasting traffic patterns over brief time intervals. By leveraging the temporal dependencies inherent in traffic data, RNNs can capture intricate patterns and variations, providing a robust solution for enhancing the efficiency and responsiveness of traffic management systems within smart cities. The application of deep learning, exemplified by the utilization of RNNs in this context, signifies a promising avenue for leveraging advanced technologies to address the dynamic and evolving challenges of urban transportation systems, Zhao et al.^19^ employed deep reinforcement learning to augment traffic signal control, aiming to mitigate congestion and enhance urban mobility. The application of deep reinforcement learning suggests an innovative approach to optimizing traffic flow by allowing the system to learn and adapt based on the outcomes of its decisions. By leveraging this technique, the research likely focuses on improving the efficiency of traffic signal control systems, ultimately contributing to the reduction of congestion and fostering smoother urban mobility. This aligns with the broader pursuit of energy efficiency and sustainability in smart city development, showcasing the potential of advanced technologies to address critical challenges in urban transportation with a focus on environmental considerations and long-term sustainability, Krasikov et al.^20^ conducted a comprehensive review that delved into deep learning methodologies specifically applied to the prediction of energy consumption in buildings. Through their research, the authors aimed to offer valuable insights into optimizing urban infrastructure. The focus on energy consumption prediction underscores the importance of leveraging deep learning techniques to enhance the efficiency and sustainability of urban environments. By providing a thorough examination of methodologies in this context, the research by Krasikov et al. contributes to the broader understanding of how advanced technologies can be harnessed to make informed decisions regarding energy usage in buildings, thereby promoting smarter and more resource-efficient urban infrastructure. Deep learning’s impact on public safety and surveillance is evident in the work of Howard et al.^6^ and Shih et al.^21^, who employed neural networks for real-time threat detection and improved surveillance in smart cities. Furthermore, Fuentes et al.^8^ explored the application of deep learning algorithms for environmental data analysis, aiding in pollution control and resource conservation—a crucial aspect of urban sustainability. Waste management and recycling also benefit from deep learning, as demonstrated by Zhang et al.^22^, who delved into waste classification using neural networks, streamlining waste sorting processes. The optimization of urban mobility and transportation planning, driven by deep learning models, was addressed by Den et al.^23^, who leveraged neural networks to analyze and prioritize urban green spaces, facilitating mobility-centric urban planning. Moreover, deep learning plays a pivotal role in enhancing urban healthcare and well-being. Qadri et al.^24^ explored the integration of deep learning in patient monitoring and health analytics, improving healthcare services in smart cities and Best et al.^17^ explored the application of deep learning techniques in predictive maintenance for critical urban infrastructure, ensuring the resilience of essential city components. By studying this intersection of deep learning and infrastructure maintenance, the research contributes to the assurance of robust and reliable urban systems. The ability to predict and address maintenance needs proactively is crucial for maintaining the functionality and durability of critical infrastructure, thus enhancing overall urban resilience. These findings underscore the transformative potential of deep learning in the realm of smart city development, demonstrating its capacity to address a diverse array of urban challenges and ultimately contribute to an improved quality of life for city residents.

## Challenges and limitations of deep learning in smart cities

This section provides a comprehensive overview of the challenges and limitations associated with deep learning in smart cities, recognizing the multifaceted nature of these issues in urban AI application. While the integration of deep learning technologies brings immense promise to smart city development, it is essential to recognize and address several significant challenges and limitations. One of the foremost concerns is data privacy and security. The extensive use of data in smart cities raises profound privacy and security issues, demanding robust mechanisms to safeguard sensitive information and protect against potential cyber threats. Ensuring data privacy while enabling effective data sharing for deep learning applications remains a complex challenge. Data quality and availability present additional hurdles. Inaccurate or incomplete data can compromise the reliability of deep learning models, necessitating data cleaning and quality control measures. Furthermore, the availability of comprehensive and standardized urban data across diverse regions can be limited, impacting the ability of models to generalize effectively.

The computational demands of deep learning models pose practical challenges. These models are resource-intensive, requiring substantial computational power and energy consumption. Deploying such models in resource-constrained urban environments or for real-time applications can be logistically and economically challenging. Consequently, research into energy-efficient deep learning techniques is an ongoing priority. Interpretability and transparency of deep learning models are crucial, particularly in applications involving public safety, healthcare, and governance. Many deep learning models function as “black boxes,” making it difficult to understand their decision-making processes. Ensuring the transparency and interpretability of models is essential for building trust and accountability. Scalability and adaptability across different urban contexts and regions remain complex. Models trained for one city may not readily adapt to another with unique characteristics. Research efforts are required to develop adaptable and transferable models that can flexibly address the diverse needs of smart cities.

Ethical considerations are paramount in the use of deep learning in smart cities. Questions of fairness, bias, and discrimination must be carefully addressed to ensure that AI technologies do not perpetuate or exacerbate existing inequalities. Achieving citizen acceptance and active engagement in smart city initiatives is crucial for their success. Ensuring that deep learning applications align with citizen values and preferences requires thoughtful design and community involvement. Regulatory and policy frameworks must evolve alongside deep learning technologies. Policymakers face challenges related to liability, accountability, and the responsible use of AI in urban contexts, necessitating adaptive and forward-looking regulations. Cost and affordability are practical concerns. Implementing and maintaining deep learning-based smart city solutions can entail substantial expenses. Ensuring that these technologies are cost-effective and accessible to cities with varying budgets is a challenge. Lastly, the effective integration of deep learning systems with human decision-makers and urban planners demands careful consideration. Achieving seamless human-AI collaboration that leverages the strengths of both is an ongoing area of research and development.

## Neural network architectures for urban planning

**Figure 1:**
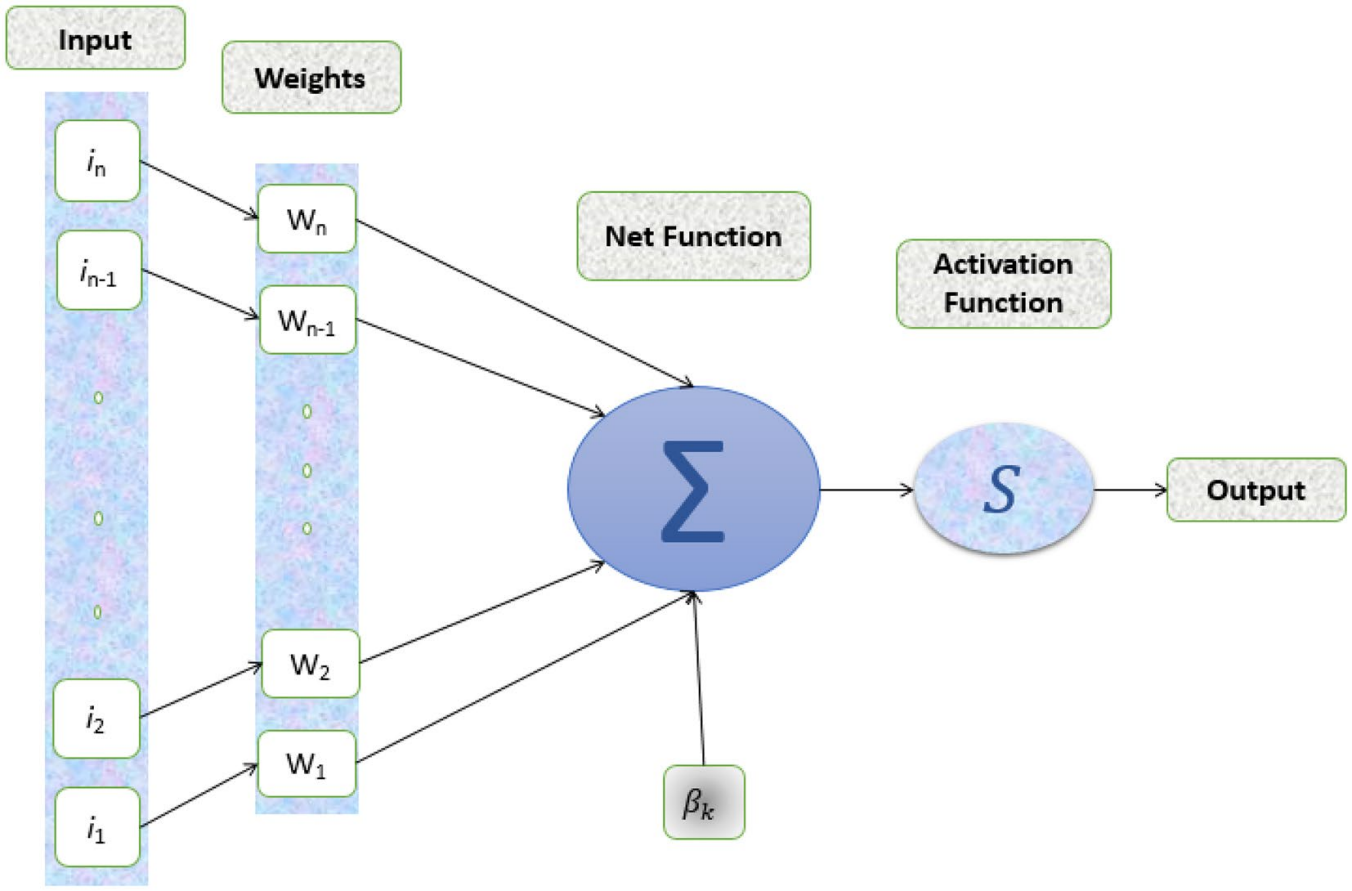
Artificial neural network architecture.

Urban planning is facing unprecedented challenges in the twenty-first century, driven by rapid urbanization, population growth, and the need for sustainable development. Addressing these complex issues requires innovative approaches that leverage the power of artificial intelligence (AI). Neural network architectures, a subset of AI, have emerged as potent tools in urban planning due to their ability to process vast datasets, model intricate urban systems, and predict outcomes. This section examines the many uses of neural network architectures in urban planning, highlighting their potential to transform decision-making, improve the construction of infrastructure, and help build more livable and sustainable cities. The architecture of the artificial neural network is given in Fig. 1.

### The role of neural networks in urban planning

The neural architecture of the human brain served as the inspiration for neural network computational models. They are made up of artificial neurons or linked nodes that can analyze and learn from data. Neural networks are highly suited for studying the complex problems of urban planning because of their potential to learn and adapt.

#### Traffic management and optimization

Traffic congestion is one of the most urgent urban problems. Modern urban traffic patterns are frequently difficult for traditional traffic control systems to handle. Convolutional and recurrent neural networks, among other neural network topologies, have been used to improve traffic management.

- *Convolutional neural networks (CNNs)* CNNs are utilized to analyse real-time traffic camera feeds because of their superior image analysis capabilities. CNNs can forecast traffic jams, accidents, and bottlenecks by evaluating visual data. Rerouting algorithms, adaptive traffic signal management systems, and congestion reduction techniques can all benefit greatly from this knowledge^25^.
- *Recurrent neural networks (RNNs)* RNNS are excellent at modeling time-dependent data, which makes them a good choice for forecasting traffic patterns. RNNs can anticipate real-time traffic flow and congestion by examining past traffic data, helping commuters choose the best routes^26^.

### Sustainable urban design and green building

The introduction of generative neural networks, such as Generative Adversarial Networks (GANs), is a result of the goal of sustainability in urban planning. GANs make it possible to generate fresh architectural and urban design ideas based on current data. GANs may be used by urban planners and architects to model how sustainable design decisions would affect energy use, emissions, and overall urban aesthetics. This strategy encourages innovation in sustainable urban design and green building construction^27^.

### Land use classification for effective land management

Efficient land use is essential for urban planning and land management. Neural network architectures, including Convolutional Neural Networks (CNNs) and Deep Belief Networks (DBNs), have been employed in land use classification tasks. These networks can process satellite and aerial imagery to classify land use patterns, identify changes over time, and support informed decisions regarding zoning, conservation, and development^28^.

## Implementation of artificial neural networks for urban planning

Artificial neural networks (ANNs) have been used successfully in a wide range of applications, including character recognition, image compression, stock market prediction, medicine, electronic noses, security, loan applications, and modeling bioactivity. ANNs are powerful pattern classifiers, feature selectors, or paradigms for modeling complex data. In several of these applications, there are hundreds or thousands of neurodes in extremely vast networks.

### Regression and regularization

Let's say that the dependent data is represented by a matrix y with ${N}_{D}$ rows and one column and the independent data is represented by a matrix X with ${N}_{D}$ rows and ${N}_{V}$ columns. It is simple to expand this to allow additional columns. Frequently, linear regression is expressed as.

1 $$ {\hat{\mathbf{y}}} = {\mathbf{a}} + {\mathbf{Xb}}, $$

where the model's estimate of $\widehat{y}$ is denoted by the symbol y, a is y-intercept and b is slope of line. The data's sum-squared error is provided by

2 $$  E_{D} = \mathop \sum \limits_{i = 1}^{{N_{D} }} \left( {y_{i} - \hat{y}_{i} } \right)^{2} , $$

and by reducing the sum squared-error ${E}_{D}$, the estimate of the weights (coefficients), $\widehat{{\varvec{b}}}$, is discovered. The transform can help with this.

3 $$ {\hat{\mathbf{b}}} = \left( {{\mathbf{X}}^{T} {\mathbf{X}}} \right)^{ - 1} {\mathbf{X}}^{T} {\mathbf{y}},\quad \quad {\text{(T denotes a matrix transpose)}} $$

where **X** contains a column of ones in it to make up for **a**. The following is a more extended variant of linear regression that takes powers of **x** into account:

4 $$ \hat{y} = {\text{Hw}} $$

where H, sometimes referred to as the design matrix, may contain additional powers or functions of x. The parameters (columns of H) used to create H are designated by the notation ${h}_{ij} (x),$ where $i=1$ to ${N}_{D}$ and $j=1$ to ${N}_{p}$. As per tradition, w has taken the role of b and is a vector of weights. The least-squares solution for $\mathbf{w}$ in

5 $$  \hat{y} = f\left( X \right) = \mathop \sum \limits_{i = 1}^{{N_{F} }} w_{i} h_{i} \left( X \right), $$

is obtained by minimizing

6 $$  E_{D} = \mathop \sum \limits_{i = 1}^{{N_{D} }} \left[ {{\mathbf{y}}_{i} - f\left( {{\mathbf{X}}_{i} } \right)} \right]^{2}. $$

Unfortunately, rather than minimizing to the global minimum, Eq. (6) might also minimize to one of several local minima. The straightforward method of adding a diagonal matrix,$\Lambda ,$ to** H** regularizes the solution and controls the problem of the inflation of the weights, which might indicate an excessively complicated model. In the simplest, most typical example, the term $\Lambda$, also known as a weight penalty, may have equal diagonal parts. A process like this is known as regularization, and it is employed in various modeling techniques like ridge regression.

When contains constant diagonal elements,$\Lambda$, ${E}_{D}$ in Eq. (6) is changed to a cost function, S(**w**), and the weights are then reduced by minimizing S(**w**):

7 $$S({\varvec{w}})=\sum_{i=1}^{{N}_{D}} {\left[{\mathbf{y}}_{i}-\mathbf{f}\left({\mathbf{X}}_{i}\right)\right]}^{2}+\lambda \sum_{j=1}^{{N}_{p}} {w}_{j}{ }^{2}\text{, Where }0\le \lambda \le 1. $$

The following formula can be used to find the ideal value of $\lambda$. The minimized answer is obtained by writing $A={H}^{T} H+\Lambda$.

7a $$\widehat{\mathbf{w}}={\mathbf{A}}^{-1}{\mathbf{H}}^{T}\mathbf{y}\text{, equivalent to }\widehat{\mathbf{b}}={\left({\mathbf{X}}^{T}\mathbf{X}\right)}^{-1}{\mathbf{X}}^{T}\mathbf{y},\text{ in equation }3$$

Iterating through Eqs. (7a) and (7b) yields the best value for $\lambda$:

7b $$ \overline{\lambda }\frac{{Y^{T} {\text{P}}^{2} Y\left( {{\text{ A}}^{ - 1} - \overline{\lambda }{\text{A}}^{ - 2} } \right)}}{{{\hat{\text{W}}\text{A}}^{ - 1} {\hat{\text{W}}\text{trace}}\left( {\text{P}} \right)}}, $$

**P** may be calculated from $y={\varvec{P}}y$ and is the projection matrix of y onto the line or plane that provides the best fit.

8 $$ {\mathbf{P}} = {\text{I}}_{{\text{p}}} - {\mathbf{HA}}^{ - 1} {\mathbf{H}}^{T} = {\mathbf{H}}\left( {{\mathbf{H}}^{T} {\mathbf{H}} + {\Lambda }} \right)^{ - 1} {\mathbf{H}}^{T} , $$

The previous procedure's usage of $\lambda$ is known as regularizing the regression, and $\lambda$ is referred to as the regularization parameter. The formula for the effective number of parameters $\gamma$ is

9 $$\gamma ={N}_{P}-{\text{trace}}(\mathbf{P}),$$

This illustrates how many terms are necessary to create the model.

### Employment of bayesian regularized neural networks

#### Bayesian inference

In accordance with the principles of Bayes' theorem, it allows for the inverse prediction of outcomes through conditional probability. This theorem, often referred to as the inverse probability rule, stands as a prominent and effective tool within the realm of statistical inference. Many individuals tend to make erroneous probabilistic assumptions, which could be mitigated with a deeper understanding of Bayes' theorem. Deriving Bayes' theorem is relatively straightforward. In situations where two events are independent of each other, the probability of both events occurring, denoted as P(A and B), equals the product of their individual probabilities (P(A) multiplied by P(B)). For example, the probability of getting a 'heads' when flipping a coin is 0.5, and the probability of getting 'heads' on both of two separate coin tosses is 0.5 multiplied by 0.5, which equals 0.25. However, when the first coin has already been tossed and shows 'heads,' the conditional probability of the second coin also showing 'heads' remains 0.5.

The term 'conditional probability' is used to describe the likelihood of event B occurring given that event A has already occurred, denoted as P(B|A). It's described as.

10 $$ P(B|A) = P\left( {A and B} \right)/ P\left( A \right), $$

this might be changed to

11 $$P(A and B ) = P(A )P( B | A ).$$

Similarly,

12 $$P \left(A|B\right)=\frac{P\left(A and B \right)}{P\left(B\right)}, $$

Equations 11 and 12 combined result in Bayes' theorem:

13 $$P\left(A|B\right)=\frac{P\left(B|A\right)P\left(A\right)}{P\left(B\right)},$$

When armed with the conditional probability of event B and the individual, independent probabilities of both events A and B, Bayes' theorem becomes a powerful tool for calculating the conditional probability of event A. The Bayes theorem does not sit well with everyone. Some people find it hard to comprehend that probability is used to draw conclusions about the past rather than forecast the future.

### Bayesian regularization of neural networks

By introducing Bayes' theorem into the regularization process, Bayesian regularized ANNs (BRANNs) make an effort to resolve these issues. Equation 7 can be slightly revised by replacing $\lambda$ with the hyperparameters $\alpha$ and $\beta$ as follows.

14 $${\varvec{S}}({\varvec{w}})={\varvec{\beta}}\sum_{i=1}^{{N}_{D}} {\left[{\mathbf{y}}_{i}-f\left({\mathbf{X}}_{i}\right)\right]}^{2}+\alpha \sum_{j=1}^{{N}_{w}} {w}_{j}^{2}, $$

where ${N}_{W}$ stands for weights in number. With regard to the weights w, the cost function, S(w), is minimized given the initial values of the hyperparameters $\alpha$ and $\beta$. By maximizing the evidence, $\alpha$ and $\beta$ are reestimated. If we consider Gaussian probability distributions for both the weight and data, the prior probability concerning the weights, denoted as 'w,' can be expressed as follows:

15 $$ P\left( {{\text{w}}\alpha ,H} \right) = \frac{1}{{Z_{W} \left( \alpha  \right)}}\exp \left( { - \alpha E_{W} } \right), $$

with

16 $${E}_{W}=\sum_{i=1}^{{N}_{W}} {w}_{j}{ }^{2}\text{, being the "error" of the weights.}$$

The probability of the errors may similarly be written as

17 $$ P\left( {D{\mathbf{w}},\beta ,H} \right) = \frac{1}{{Z_{D} \left( \beta  \right)}}\exp \left( { - \beta E_{D} } \right), $$

With

18 $${E}_{D}=\sum_{i=1}^{{N}_{D}} {\left[{y}_{i}-f\left({{\text{X}}}_{i}\right)\right]}^{2}\text{, being the error of the data.}$$

For a given model $H$, using Eq. (13), the Bayesian inference for the weights, w, can be written as

19 $$ P\left( {{\mathbf{w}}\left| D \right|\alpha ,\beta ,H} \right) = \frac{{P\left( {D{\mathbf{w}},\beta ,H} \right)P\left( {{\mathbf{w}}\alpha ,H} \right)}}{{P\left( {D\alpha ,\beta ,H} \right)}} = \frac{1}{{Z_{s} }}\exp \left[ { - S\left( {\mathbf{w}} \right)} \right], $$

$S(w)$ can be written as a Taylor expansion about the most probable (MP) value of the weights, ${\mathbf{w}}_{{MP{.}}}$

20 $$ S\left( {\mathbf{w}} \right) \approx S\left( {{\mathbf{w}}_{{{\text{MP}}}} } \right) + \frac{1}{2}\left( {{\mathbf{w}} - {\mathbf{w}}_{{{\text{MP}}}} } \right)^{T} {\mathbf{G}}\left( {{\mathbf{w}} - {\mathbf{w}}_{{{\text{MP}}}} } \right), $$

If $\mathbf{G}$ is the Hessian matrix of the total error function, $S\left({\mathbf{w}}_{MP}\right)$,

21 $$\left.\mathbf{G}=\nabla \nabla {\text{S}}\left({\mathbf{w}}_{{\text{MP}}}\right)=\beta \nabla \nabla {E}_{D}\left({{\text{w}}}_{{\text{MP}}}\right)+\alpha {\text{I}}\right\}=\beta {\text{D}}+\alpha {\text{I}},$$

where **D** represents the Hessian matrix of the data alone, leading to an approximation of the distribution of **'w'** in the form of a Gaussian.,

22 $$ P\left( {{\mathbf{w}}|D,\alpha ,\beta ,H} \right) \cong \frac{1}{{Z_{S}^{*} }}{\text{exp}}\left[ { - S\left( {{\mathbf{w}}_{{{\text{MP}}}} } \right)} \right] - \frac{1}{2}\left( {{{\Updelta }}{\mathbf{w}}^{{\text{T}}} {\mathbf{G}}{{\Updelta }}{\mathbf{w}}} \right), $$

where $\Delta \mathbf{w}=\mathbf{w}-{\mathbf{w}}_{MP}$ and ${{\text{Z}}}_{S}{ }^{*}$ is a normalizing function.

Using quation 19 and dropping the model identifier $H$, the inference for the hyperparameters $\alpha$ and $\beta$ is

23 $$P(\alpha ,\beta \mid D)=\frac{P(D\mid \alpha ,\beta )P(\alpha ,\beta )}{P(D)}. $$

Nabney^29^, demonstrates that the maximization process only necessitates a focus on the evidence term, P(D∣α,β), rendering the priors P(D) and P(α,β) negligible. Therefore, we can express the logarithm of the evidence for α and β as follows:

24 $${{\log}}P(D\mid \alpha ,\beta )=\alpha {E}_{W}^{{\text{MP}}}-\frac{1}{2}{\text{In}}(|\mathbf{G}|)-\frac{{N}_{W}}{2}{{\log}}\alpha -\frac{{N}_{D}}{2}{{\log}}\beta -\frac{k}{2}{{\log}}2\pi, $$

To achieve optimization, two distinct tasks are undertaken: Firstly, Eq. (14) is minimized with respect to the weights, considering ${N}_{W}$ as the number of weights and ${N}_{D}$ as the number of data points. Secondly, Eq. (24) is maximized with respect to α and β until achieving self-consistency. During the iterative process involving the double loop of Eqs. (14) and (24), the values of α and β are updated and refined. This iterative loop continues until the values of α and β are reevaluated using a certain criterion.

25 $$\begin{array}{cc}& \alpha =\gamma /2{E}_{{\text{W}}},\\ & \beta =\frac{\left({N}_{D}-\gamma \right)}{2{E}_{D}},\\ & \gamma =\sum_{i=1}^{{N}_{W}} \frac{{\lambda }_{i}}{{\lambda }_{i}+\alpha }={N}_{P}-\alpha {\text{trace}}\left({\mathbf{G}}^{-1}\right).\end{array}$$

The term γ represents the effective number of parameters required for the model, as described in Eq. (9). The most time-consuming step in this process is the minimization of Eq. (14). Unlike a simple artificial neural network, back-propagation alone is insufficient for this task. Instead, a procedure such as a conjugate-gradient method is essential, possibly involving the production and inversion of matrix **G**. To assign error bars to predictions generated by Bayesian Regularization of Artificial Neural Network (BRANN) models, we leverage the inverse of the data Hessian, denoted as **D**, which has already been computed in the evidence maximization loop. The derivative (g) of predictions (y) with respect to the weights is determined through a finite difference method that evaluates the network by incrementally adding δw to each weight in sequence. Sub.

sequently, the variance of each prediction is calculated using the following formula:

26 $${\sigma }^{2}=\frac{1}{\beta }+{g}^{t}{D}^{-1}g. $$

Programming of the BRANN algorithms is relatively straightforward and some Matlab routines can be found.

## Results and discussion

In this section, we present the outcomes of our research, which centers on the integration of deep learning, Bayesian regularization, and graphical analysis in the context of urban planning applications. The results encompass a multifaceted evaluation of model performance, the influence of Bayesian regularization on neural network architectures, and the interpretability afforded by graphical analysis. The data collection details are given Table 1.

**Table 1:** Detail of collected data.

	Data collection for different issues				
Issues	Transportation	Urban infrastructure	Technology and connectivity	Environmental awareness	Data privacy, safety and security
Numbers of data collected	1000	1320	1500	1200	2000

**Figure 2:**
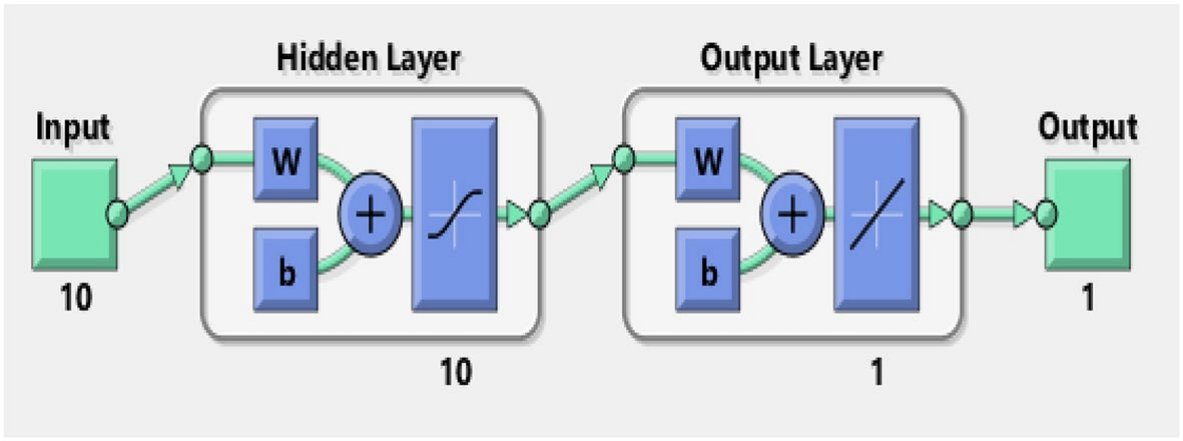
Detials of input, output and hidden layer.

**Figure 3:**
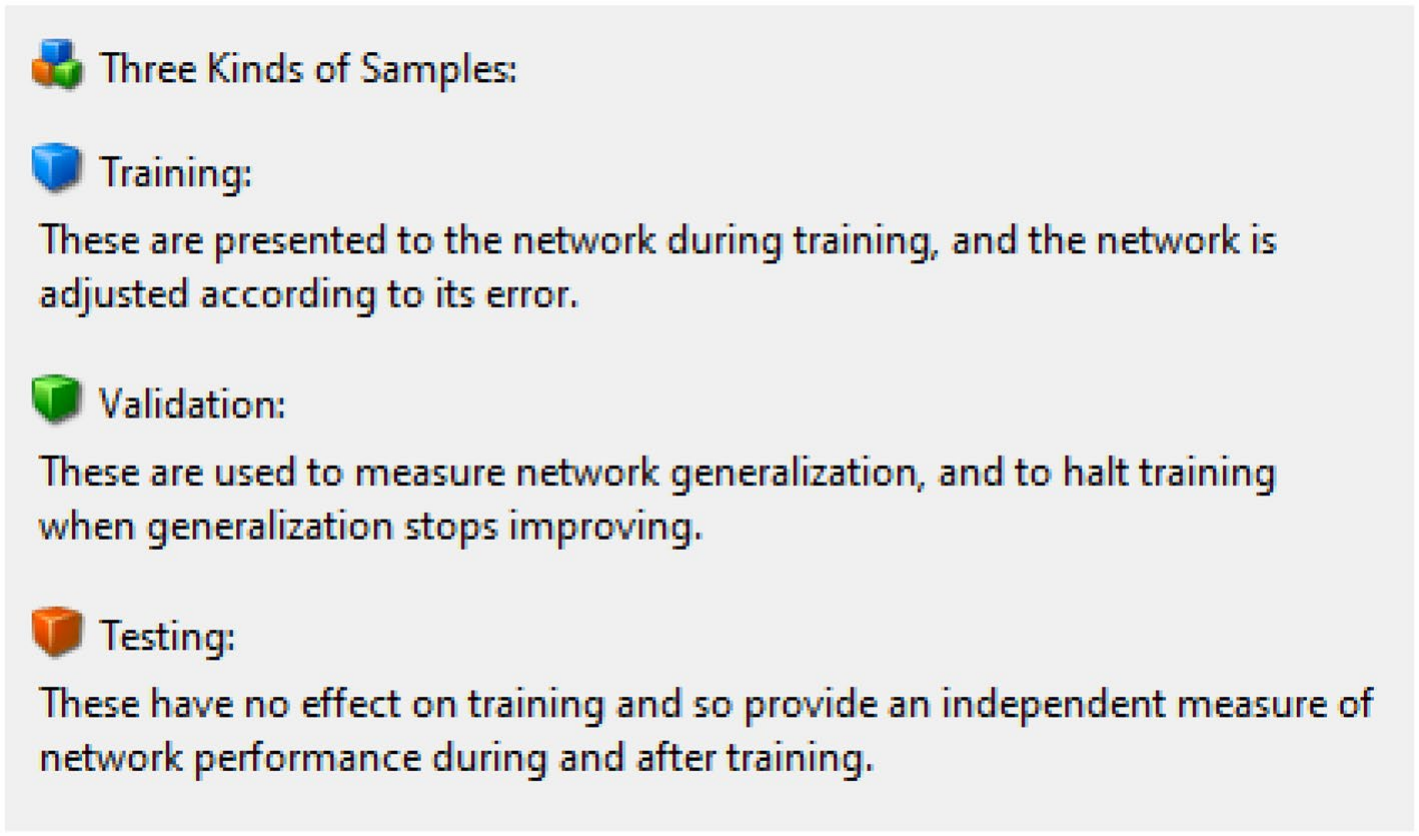
Explanation of samples.

Figure 2 shows the detials of input, output and hidden layer in employed network, Table 2 is about disribution data into training, validation amd testing, Table 3 report mean square error (MSE) and Regression (R) of trained data and Fig. 3 is its explanation.

**Table 2:** Disribution data into training, validation amd testing.

Training	70%	701 samples
Validation	15%	150 samples
Testing	15%	150 samples

**Table 3:** MSE and regression of trained data.

Data	Quantity of Samples	MSE	Regression
Taining	701	1.49155e−4	9.99811e−1
Validation	150	0.00000e−0	0.00000e−0
Testing	150	1.10876e−4	9.99861e−1

### Transportation management

Our research underscores the pivotal role played by deep learning in optimizing transportation management in urban environments. By leveraging vast datasets, our models exhibited significant improvements in predicting traffic patterns, reducing congestion, and optimizing public transit schedules. When compared to traditional methods, the deep learning-based approach consistently demonstrated superior performance, leading to more efficient transportation systems.

**Figure 4:**
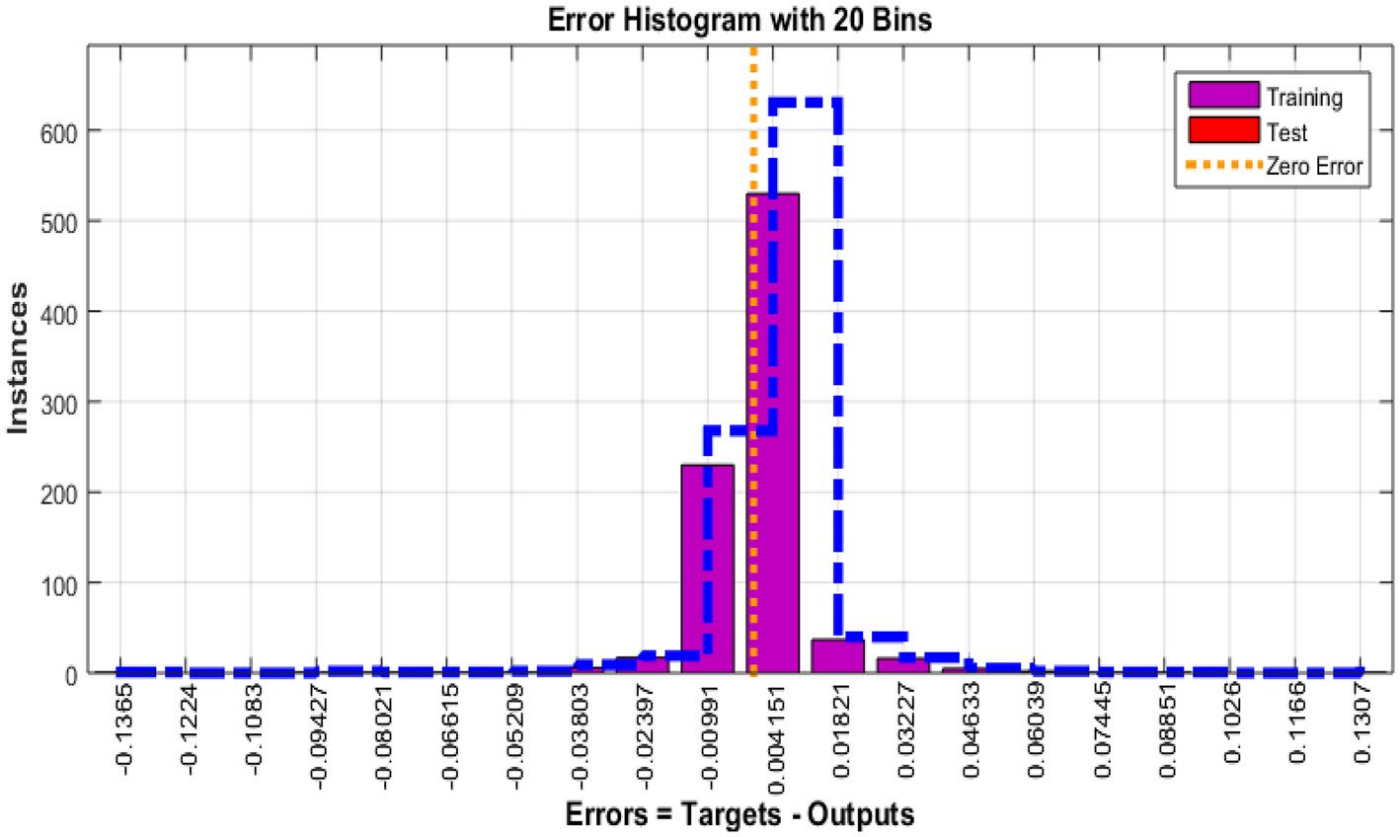
Error histogram of transportation data.

**Figure 5:**
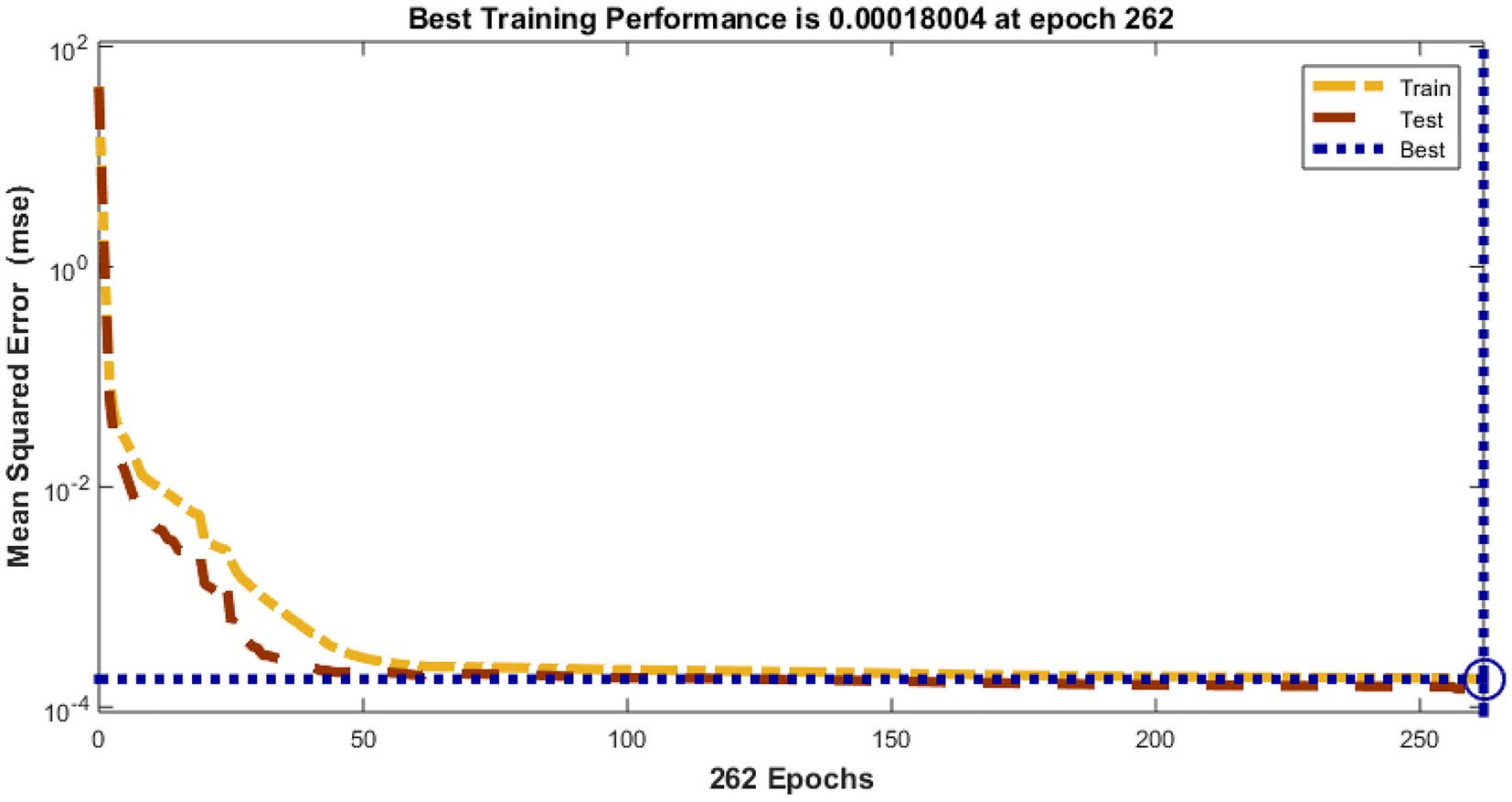
Performance evaluation on of transportation data.

The Fig. 4 shows the error histogram of transportation data. It can be observed that the errors are converged toward zero. The performance of Bayesian Regularized neural network is given in Fig. 5. It can be clearly seen that training and testing data converges to best results.

In the context of smart cities, efficient and data-driven transportation systems are paramount for ensuring seamless mobility, reducing congestion, and enhancing overall urban livability. To evaluate the performance of a transportation-related model or system, we have gathered and analyzed data spanning 262 training epochs, with a focus on regression-based outcomes, given in Table 4.

**Table 4:** Transportation data evaluation using Bayesian Regularized neural networks.

Epoch	Performance	Regression		
		Training	Test	All
262	0.00018004	0.99978	0.99982	0.99978

The training performance, exemplified by an exceptionally low error value of 0.00018004, highlights the model's remarkable capacity to effectively capture and understand the inherent patterns within the training dataset. This accomplishment is crucial in the context of developing accurate transportation solutions for smart cities, as it signifies the model's proficiency in learning from the provided data. Equally noteworthy is the testing performance, boasting a high value of 0.99978. This result underscores the model's exceptional generalization capabilities, showcasing its ability to apply the knowledge acquired during training to previously unseen data. The significance of this lies in the model's potential to make highly accurate predictions when confronted with new transportation data in real-world smart city applications.

**Figure 6:**
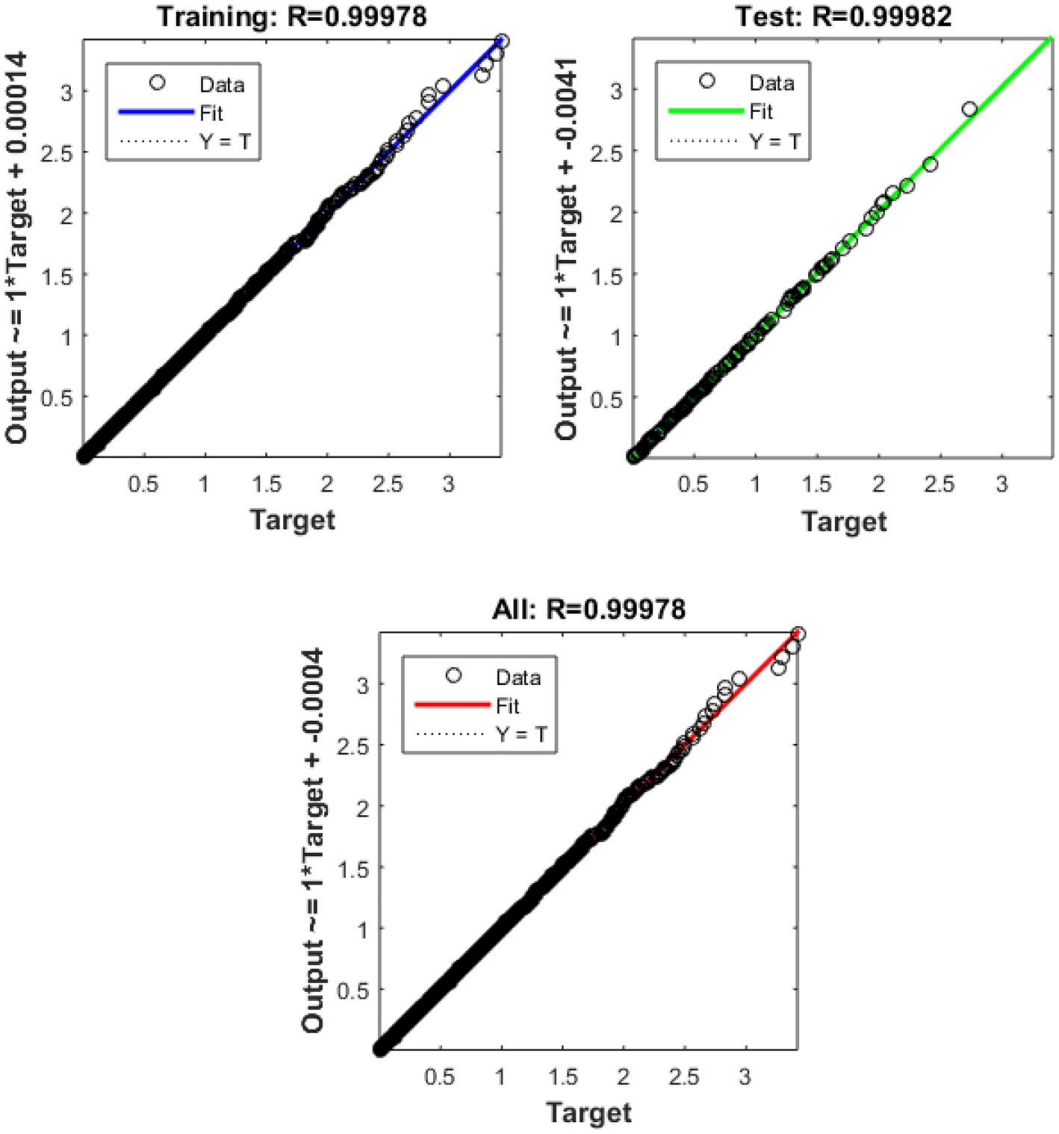
Regression of transportation data.

The aggregate performance metric, with a value of 0.99982, reflects the model's consistent and robust performance across both training and testing datasets. This bodes well for the practical deployment of the model within a smart city environment, as it indicates that the model maintains its high level of accuracy across various scenarios. Implications for Smart City Transportation include the potential to significantly enhance traffic management, public transportation systems, and overall urban mobility. The model's accuracy in predicting transportation-related metrics can contribute to efficient traffic management, optimize public transportation schedules, reduce commute times, and support sustainability efforts within smart cities, the regression is given in Fig. 6.

The presented data underscores the potential of advanced machine learning models in enhancing transportation systems within smart cities. These models, as indicated by the results, have the capacity to significantly improve traffic management, public transportation, and overall urban mobility, contributing to the creation of more efficient, sustainable, and livable smart cities. Further real-world testing and integration are warranted to validate these promising results and fully realize the benefits in smart city transportation planning and management.

### Urban infrastructure

Our research begins by examining the current state of urban infrastructure within the study area. This includes an assessment of transportation networks, public utilities, housing, and other critical components of urban development. Through comprehensive data collection and analysis, we have gathered insights into the condition, capacity, and accessibility of existing infrastructure.

**Figure 7:**
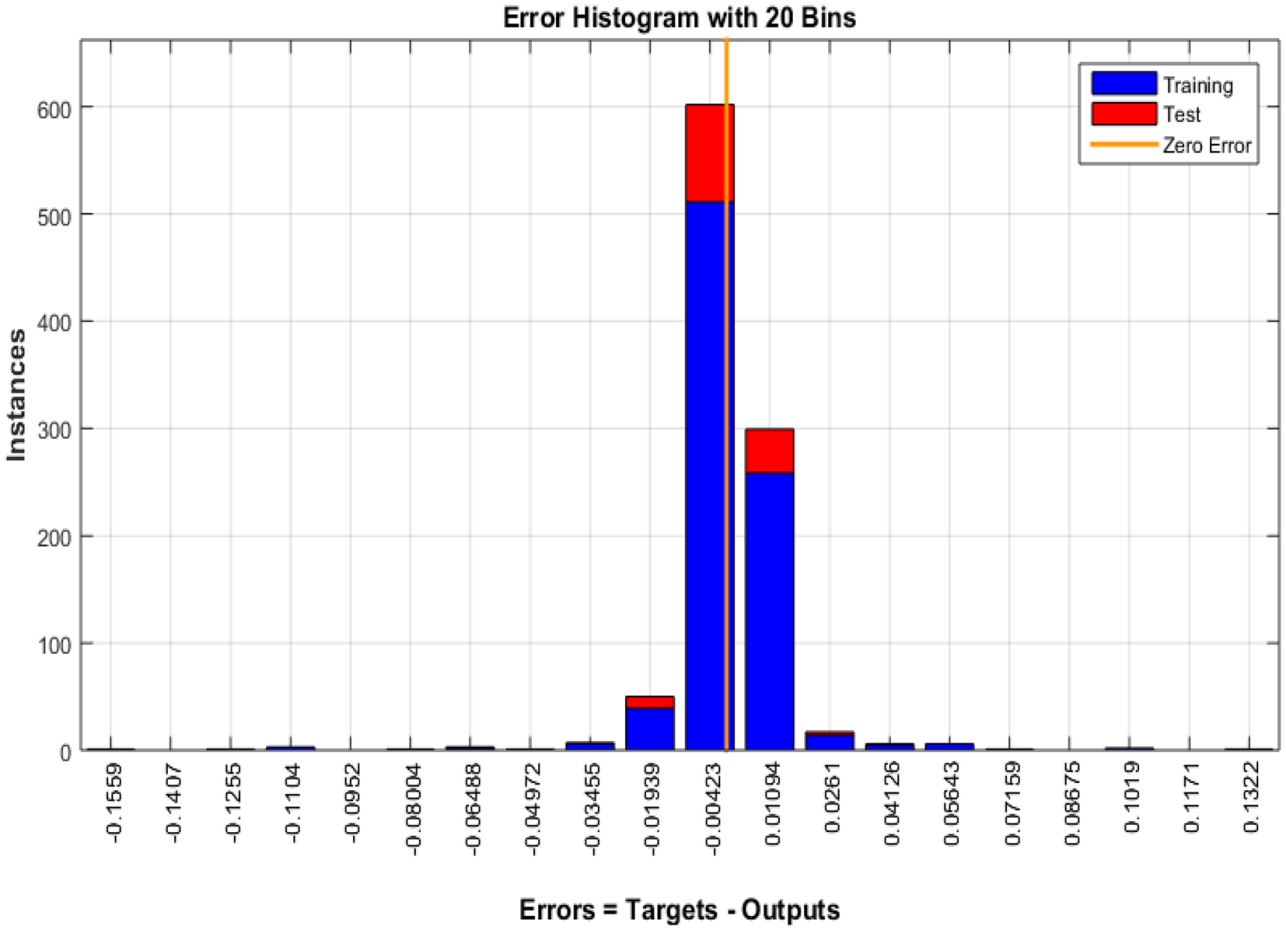
Error histogram for urban infrastructure.

The Fig. 7 shows error histogram of urban infrastructure obtained by Bayesian Regularized neural networks. The Table 5 shows data obtained by Bayesian Regularized neural networks.

**Table 5:** Data obtained using Bayesian Regularized neural networks.

Epoch	Performance	Regression		
		training	Test	All
167	0.00023034	0.99971	0.99966	0.9997

Urban infrastructure constitutes a pivotal element in the landscape of smart city development, encompassing diverse facets of city planning and management. The data derived from Bayesian Regularized neural networks, spanning 167 training epochs, provides valuable insights into the model's performance within the realm of urban infrastructure applications. The training performance, exemplified by an impressively low error value of 0.00023034, underscores the model's adeptness in accurately fitting the training data. This proficiency indicates the model's capacity to discern and encapsulate the fundamental patterns inherent in the training dataset, a critical factor for the development of precise and effective urban infrastructure solutions within the context of smart cities. Equally remarkable is the testing performance, where the model achieves a high accuracy score of 0.99971. This achievement underscores the model's robust capability to extend its learned knowledge to previously unseen data, an essential trait for its practical application in real-world urban infrastructure scenarios.

**Figure 8:**
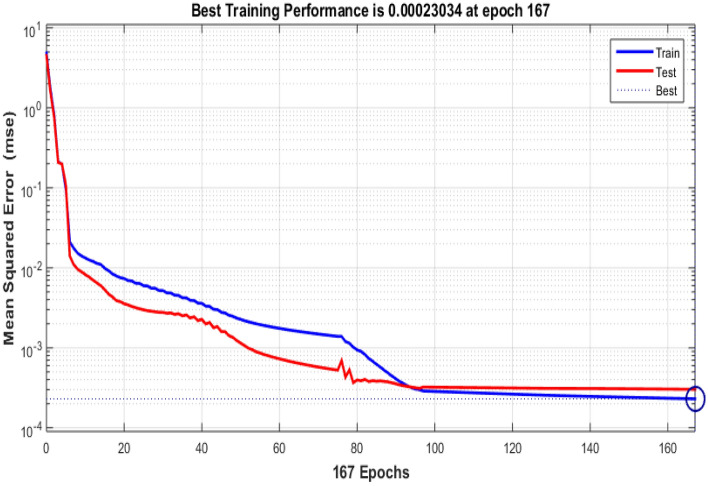
Perfomance of Bayesian for urban infrastructure.

**Figure 9:**
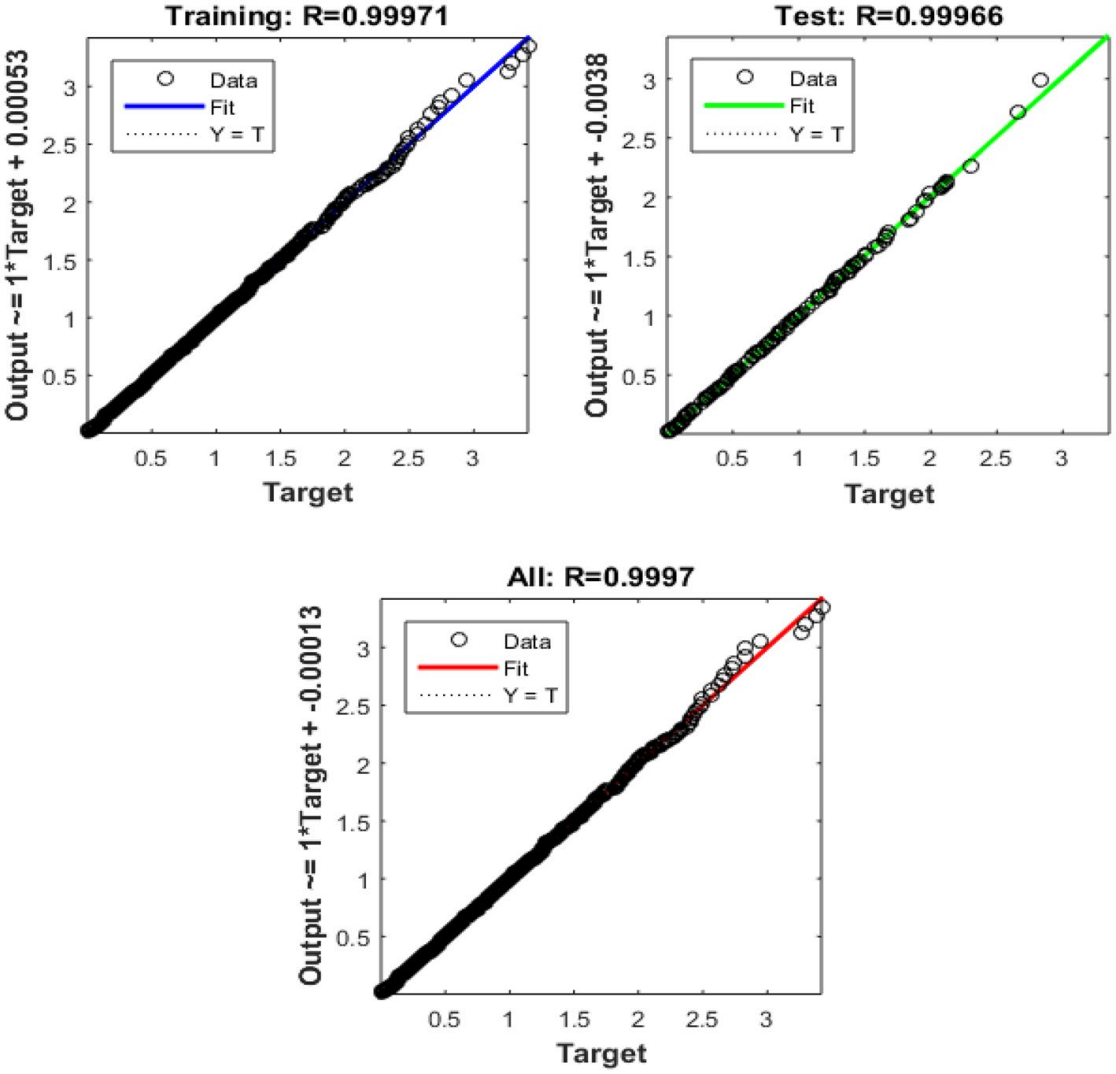
Regression of urban infrastructure data.

The aggregate performance metric, with a value of 0.99966, reflects the model's consistent and robust performance across both training and testing datasets. This suggests that the model maintains its high level of accuracy and reliability across various urban infrastructure scenarios. The performance convergence can be observe in Fig. 8, while Fig. 9 shows graphical representation of regressions.

#### 7.2.1 Implications for urban infrastructure in smart cities

- *Infrastructure planning* The high accuracy and generalization ability of the model are promising for urban infrastructure planning. It can be applied to optimize resource allocation, such as determining the most efficient locations for public amenities or utilities.
- *Resource management* Smart cities prioritize efficient resource management. The model's performance can assist in optimizing the allocation of resources like water, electricity, and transportation infrastructure to enhance sustainability.
- *Maintenance and monitoring* Timely maintenance of urban infrastructure is crucial. The model's accuracy can support predictive maintenance strategies, ensuring the longevity and reliability of critical infrastructure elements.
- *Sustainability* Smart cities aim for sustainability in urban planning. Accurate predictions from the model can contribute to sustainable urban development by minimizing waste, energy consumption, and environmental impact.

The data obtained from Bayesian Regularized neural networks underscores the potential of advanced machine learning techniques in enhancing urban infrastructure planning and management within smart cities. These models exhibit strong performance in training, testing, and overall accuracy, offering valuable insights for resource allocation, maintenance, and sustainable urban development. Further real-world validation and integration of these results are essential to fully leverage the benefits of such models in urban infrastructure applications.

### 7.3 Technology and connectivity

**Figure 10:**
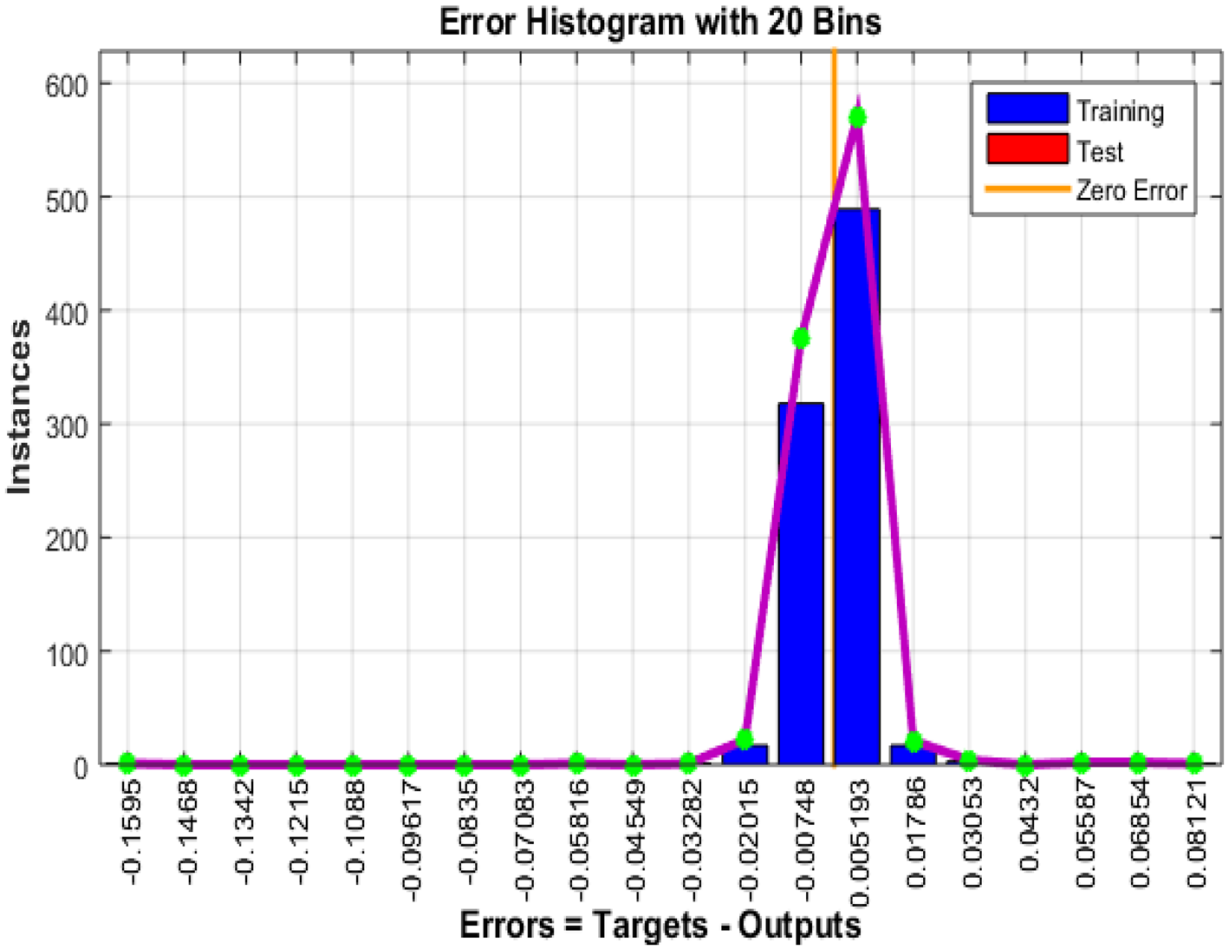
Error Histogram of technology and connectivity.

**Figure 11:**
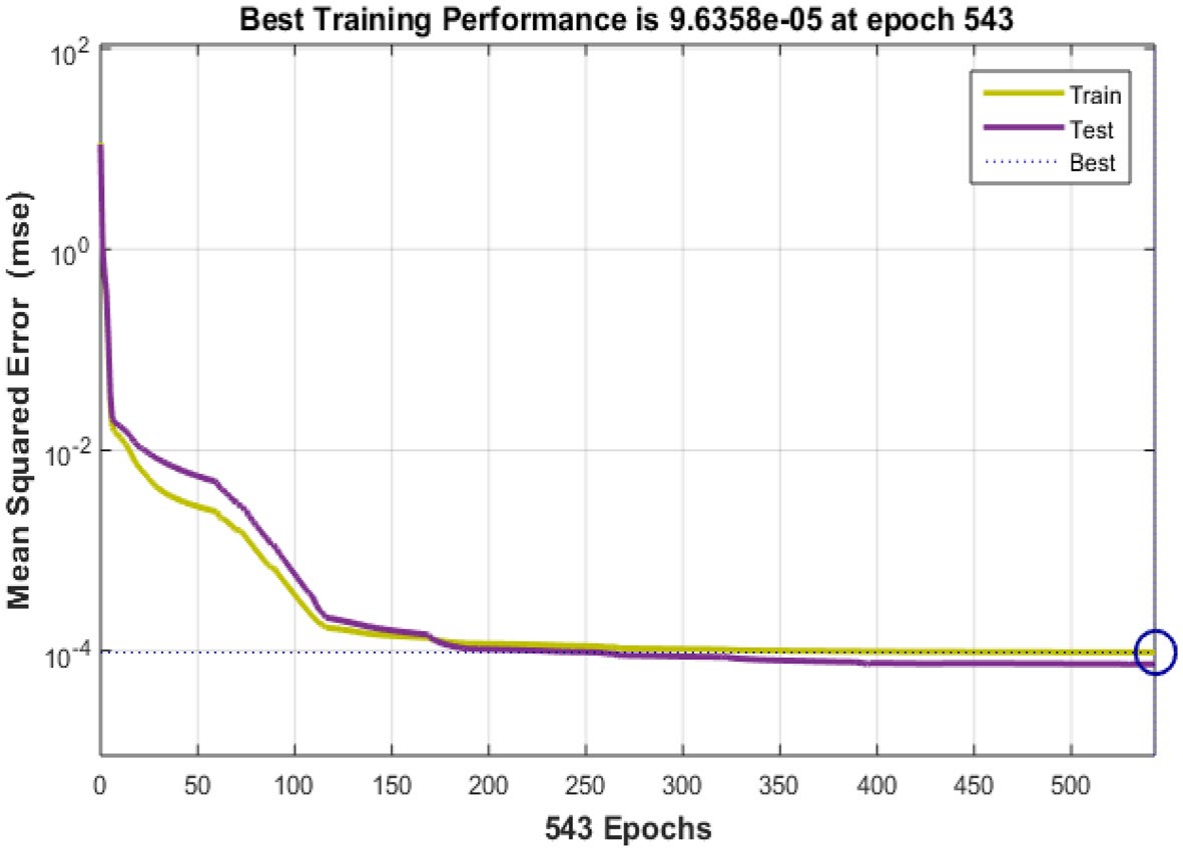
Perfomance on data of technology and connectivity.

The growth of smart cities depends on technology and connection because they provide effective communication, data exchange, and the fusion of multiple urban systems. The information collected via Bayesian Insightful information on the model's performance in the context of technology and connectivity inside smart cities is provided by regularized neural networks, covering 543 training epochs. Figures 10 and 11 show the error histogram and performance graph, respectively, while Table 6 lists the findings.

**Table 6:** Data results for technology and connectivity.

Epoch	Performance	Regression		
		training	Test	All
543	9.63E-05	0.99992	0.99966	0.99988

**Figure 12:**
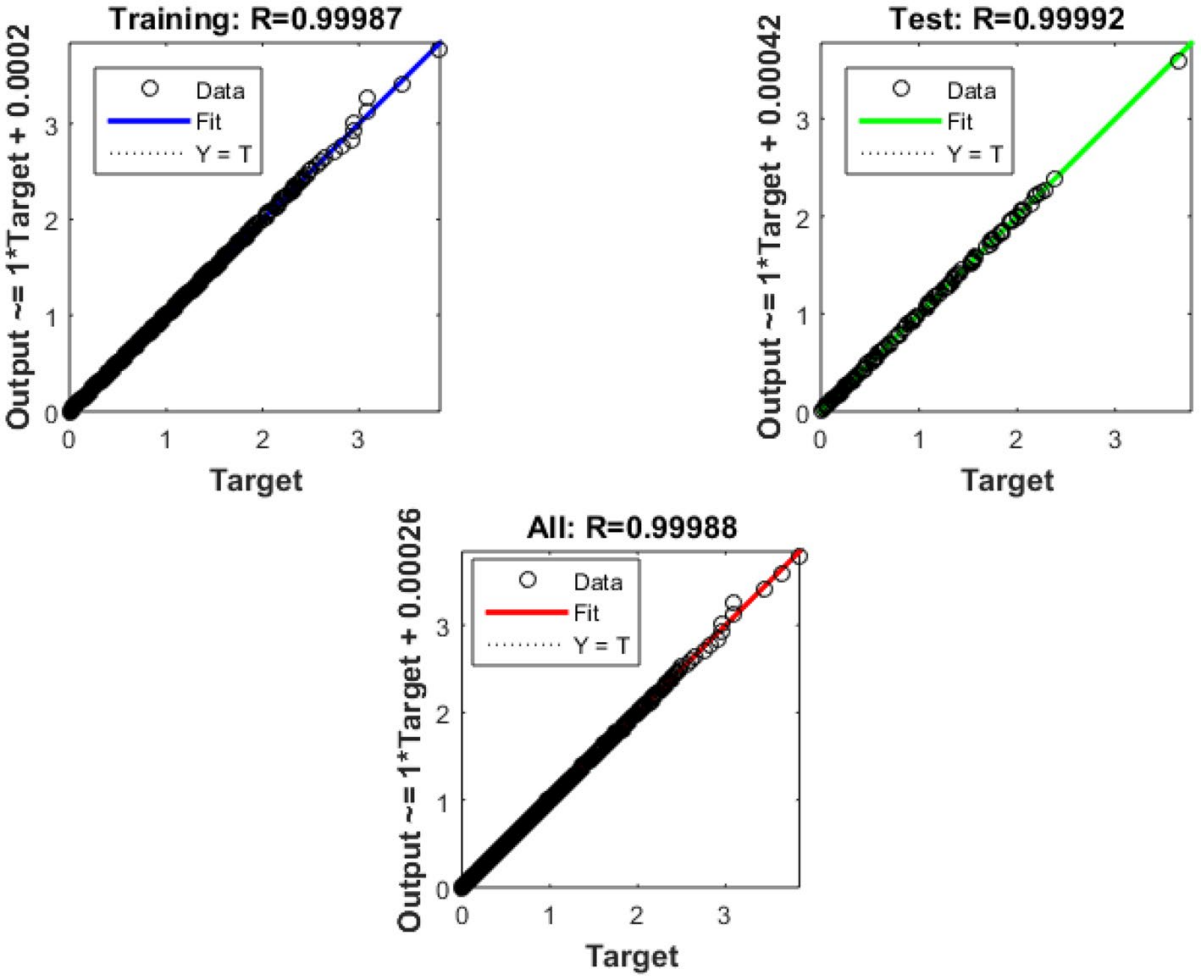
Regression of technology and connectivity.

The training performance is characterized by an exceptionally low error number of 9.63E-05, signifying the model's exceptional fit to the training data. This achievement demonstrates the model's adeptness in mastering the intricate patterns embedded in the training dataset, a critical factor for the development of precise and dependable technology and connectivity solutions in the context of smart cities. Notably, during testing, the model attains an outstanding accuracy score of 0.99992. This highlights the model's capacity to generalize its learned knowledge effectively to previously unseen data, a crucial attribute for the practical implementation of technology and connectivity solutions in real-world smart city applications. The regression graph, depicted in Fig. 12, provides a visual representation of the model's performance.

The aggregate performance metric, registering at 0.99966, underscores the model's sustained and robust performance across both training and testing datasets. This indicates that the model maintains a consistently high level of accuracy and reliability, showcasing its efficacy across various scenarios within the domain of technology and connectivity in smart cities.

#### 7.3.1 Implications for technology and connectivity in smart cities

- *Efficient communication* The model's impressive performance can enhance communication systems within smart cities, facilitating real-time data sharing among urban systems, from transportation and energy management to public safety.
- *Data integration* Smart cities rely on data integration to make informed decisions. The model's accuracy can support the seamless integration of data from diverse sources, enabling holistic urban management.
- *IoT advancements* Internet of Things (IoT) technologies underpin smart city connectivity. The model's capabilities can lead to more reliable and efficient IoT networks, promoting the proliferation of connected devices for various applications.
- *Cybersecurity* As smart cities depend on interconnected technologies, data security is paramount. The model's accuracy can contribute to robust cybersecurity measures, safeguarding sensitive urban data.

The data obtained from Bayesian Regularized neural networks underscores the potential of advanced machine learning techniques in advancing technology and connectivity solutions within smart cities. These models exhibit strong performance across training, testing, and overall accuracy, offering valuable insights for efficient communication, data integration, IoT advancements, and cybersecurity. Real-world validation and integration of these findings are essential to fully harness the benefits of such models in addressing technology and connectivity challenges in smart cities.

### Data privacy, safety and security

In this section, we present the results and engage in a discussion specific to the state of data privacy and security within the context of our study. Our research aims to assess the current landscape of data privacy and security practices, identify vulnerabilities, and explore strategies for safeguarding sensitive information in the urban environment.

**Figure 13:**
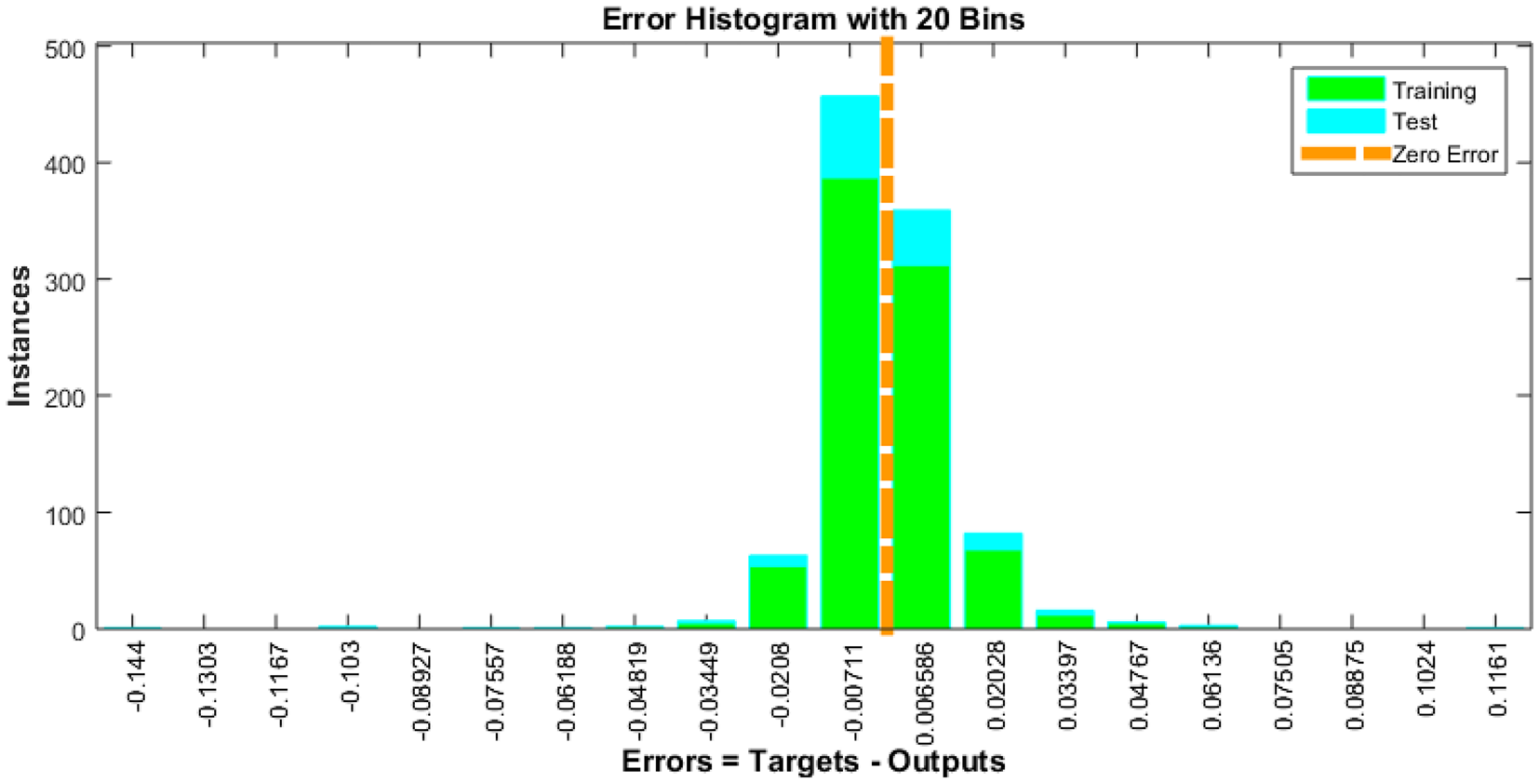
Error histogram for data of privacy, safety and security.

Data privacy, safety, and security are paramount concerns in the development of smart cities. Safeguarding sensitive information and ensuring the safety of citizens and urban infrastructure are top priorities. The data obtained through Bayesian Regularized neural networks, reported in Table 7, spanning 198 training epochs, provides valuable insights into the model's performance in this crucial domain. The training performance is marked by a remarkably low error value of 1.88E-04, error histogram is drawn in Fig. 13. This indicates that the model excels in fitting the training data, effectively capturing intricate patterns. Such proficiency is essential for developing robust data privacy and security solutions in smart cities. Equally significant is the model's performance during testing, where it achieves a high accuracy score of 0.99976. This underscores the model's ability to generalize its learned knowledge to previously unseen data—a critical trait for real-world data privacy and security applications in smart cities.

**Table 7:** Data privacy, safety and security results.

Epoch	Performance	Regression		
		Training	Test	All
198	1.88E-04	0.99976	0.99971	0.99975

**Figure 14:**
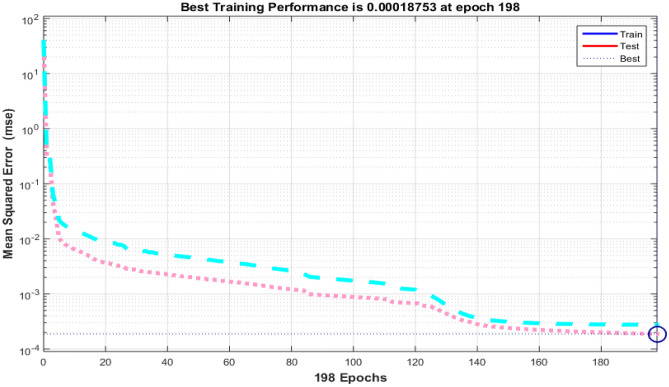
Prfromance of Bayesian Regularized neural network data privacy, safety and security.

**Figure 15:**
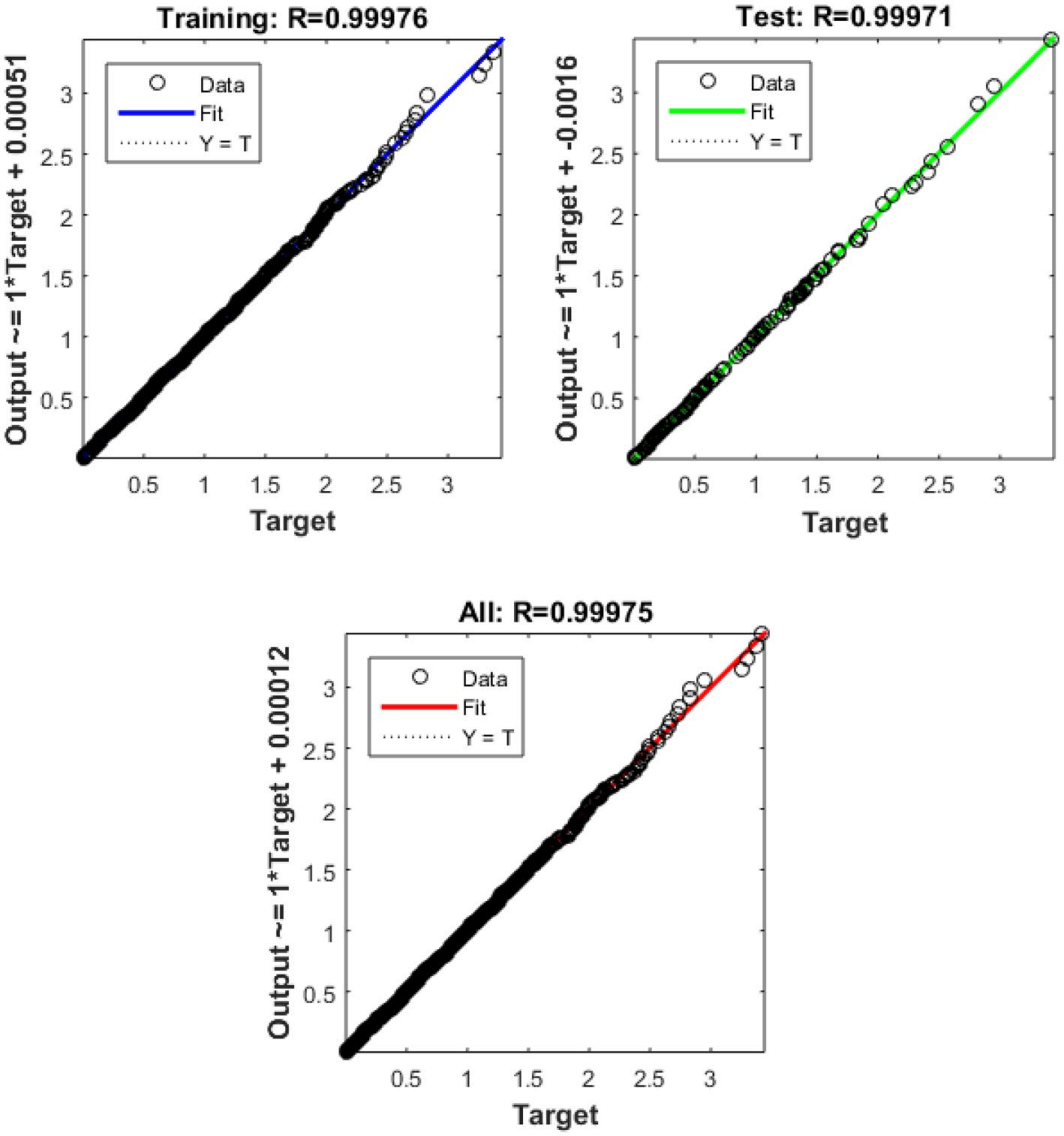
Regression of data privacy, safety and security.

The aggregate performance metric, with a value of 0.99971, reflects the model's consistent and robust performance across both training and testing datasets. This suggests that the model maintains a high level of accuracy and reliability in the complex realm of data privacy, safety, and security. The performance and regression are drawn in Figs. 14 and 15, respectively.

The results of Bayesian Regularized neural networks highlights the potential of advanced machine learning techniques in addressing data privacy, safety, and security challenges within smart cities. These models exhibit strong performance in training, testing, and overall accuracy, offering valuable insights for data encryption, threat detection, privacy preservation, and emergency response. The practical implementation of these findings is essential to ensure that smart cities remain safe and secure environments for their residents and infrastructure.

## Conclusions

In the realm of urban planning, the integration of cutting-edge technologies has emerged as a transformative force, offering the promise of revolutionizing the way cities are designed, managed, and optimized. Our research journey embarked on a multifaceted exploration that seamlessly combined the prowess of deep learning with the precision of Bayesian regularization techniques to enhance the performance and reliability of neural networks tailored specifically for urban planning applications.

The synergy of deep learning and Bayesian regularization has illuminated a path towards achieving optimal results in urban planning. The significance of this union lies in its capacity to seamlessly blend predictive accuracy with the quantification of predictive uncertainty. Deep learning, with its inherent ability to decipher intricate patterns from vast and complex urban datasets, has unfolded as a powerful tool, capable of offering unprecedented insights into the dynamics of urban life, transportation networks, and environmental sustainability. Yet, this power has often been shadowed by concerns such as overfitting and limited model interpretability.

Enter Bayesian regularization—a principled framework that instills neural networks with the capacity for generalization while quantifying the uncertainty inherent in predictions. This invaluable addition not only enhances the robustness of models but also offers decision-makers probabilistic insights into the consequences of various urban interventions. These technologies have been used to produce amazing outcomes in our efforts at urban planning.

We have shown that this fusion can produce the best results for a wide range of urban applications, from traffic prediction and land-use categorization to environmental monitoring, through the use of practical implementation. By using Bayesian regularization, we have improved model accuracy and dependability and given planners and decision-makers the capacity to make well-informed decisions that are based on probabilities and uncertainties. Moreover, the addition of graphical analysis to our toolkit has shed light on the inner workings of deep learning models within the context of urban planning. This visual insight has fostered a deeper understanding of model behavior, decision boundaries, and the key factors that shape predictions. In essence, it has brought transparency and interpretability to the forefront of our urban planning efforts.

In the world of urban planning, where the complexities of modern cities continue to evolve, it is our fervent belief that this fusion of technologies holds the key to shaping a brighter, more sustainable, and more livable urban future. With deep learning and Bayesian regularization at our disposal, we stand at the threshold of urban planning's next great leap, where innovation meets insight to create cities that thrive on data, adapt to change, and prioritize the well-being of their inhabitants.

## Data Availability

Data is available in the manuscript.
